# Methodological procedures for priority setting mental health research: a systematic review summarising the methods, designs and frameworks involved with priority setting

**DOI:** 10.1186/s12961-023-01003-8

**Published:** 2023-06-26

**Authors:** Kris Deering, Neil Brimblecombe, Jane C. Matonhodze, Fiona Nolan, Daniela A. Collins, Laoise Renwick

**Affiliations:** 1 University of Exeter Medical School, St Luke’s Campus, 79 Heavitree Rd, Exeter, EX1 2LT United Kingdom; 2 London South Bank University, 103 Borough Road, London, SE1 0AA United Kingdom; 3 University of Greenwich, Avery Hill Campus, Southwood Site, Avery Hill Road, London, SE9 2UG United Kingdom; 4 Anglia Ruskin University, Chelmsford Campus Bishop Hall Lane, Chelmsford, CM1 1SQ United Kingdom; 5 The University of Manchester, Oxford Rd, Manchester, M13 9PL United Kingdom

**Keywords:** Research priority setting, Mental health research, Priority setting frameworks, Priority setting designs, Priority setting methods, Methodological procedures

## Abstract

**Background:**

Research priority setting aims to identify research gaps within particular health fields. Given the global burden of mental illness and underfunding of mental health research compared to other health topics, knowledge of methodological procedures may raise the quality of priority setting to identify research with value and impact. However, to date there has been no comprehensive review on the approaches adopted with priority setting projects that identify mental health research, despite viewed as essential knowledge to address research gaps. Hence, the paper presents a summary of the methods, designs, and existing frameworks that can be adopted for prioritising mental health research to inform future prioritising projects.

**Method:**

A systematic review of electronic databases located prioritisation literature, while a critical interpretive synthesis was adopted whereby the appraisal of methodological procedures was integrated into the synthesis of the findings. The synthesis was shaped using the good practice checklist for priority setting by Viergever and colleagues drawing on their following categories to identify and appraise methodological procedures: (1) Comprehensive Approach—frameworks/designs guiding the entire priority setting; (2) Inclusiveness –participation methods to aid the equal contribution of stakeholders; (3) Information Gathering—data collecting methods to identify research gaps, and (4) Deciding Priorities—methods to finalise priorities.

**Results:**

In total 903 papers were located with 889 papers removed as either duplicates or not meeting the inclusion and exclusion criteria. 14 papers were identified, describing 13 separate priority setting projects. Participatory approaches were the dominant method adopted but existing prioritisation frameworks were modified with little explanation regarding the rationale, processes for adaptation and theoretical foundation. Processes were predominately researcher led, although with some patient involvement. Surveys and consensus building methods gathered information while ranking systems and thematic analysis tend to generate finalised priorities. However, limited evidence found about transforming priorities into actual research projects and few described plans for implementation to promote translation into user-informed research.

**Conclusion:**

Prioritisation projects may benefit from justifying the methodological approaches taken to identify mental health research, stating reasons for adapting frameworks alongside reasons for adopting particular methods, while finalised priorities should be worded in such a way as to facilitate their easy translation into research projects.

## Introduction

There is urgency to prioritise mental health research and undertake studies given the scale of international mental health problems, not only in terms of rising mental illness since the Covid pandemic, but also considering the early mortality rates of approximately 20 years for people with serious mental health conditions [1, 2]. It is now recognised that the importance of mental health research is equal to other health topics, including the prioritising of mental health studies [3]. Prioritising mental health research tends to adopt multidimensional approaches given the diversity in what impacts on mental health [4]. Methodological heterogeneity is common owing to different purposes, aims and contextual factors, alongside vast agendas about which research to prioritise from estimating the magnitude of mental illness burden to identifying gaps with care delivery [5]. However, Wykes et al. [6] highlights around a 20-year gap for research to be implemented, and to address specific mental health problems in society, the targeting of research needs to improve.

The World Health Organisation (WHO) [7] describes priority setting as an interpersonal activity to identify research questions and/or topics with the greatest potential for public benefit. Priority setting may commence with the reviewing of existing studies, alongside guidelines and policies to determine knowledge gaps within a research field [7]. The importance of these gaps is then refined, and prioritised in order of importance, with ideally the top priority put forward as a research project [8]. Prioritising of mental health research is argued to take a holistic view including intersecting social issues such as unemployment and mental health seen important to patients [9]. Nevertheless, questions are raised about a bio-pharmacological focus, suggesting social issues can be overlooked as scientific views might take precedence over patients given the social standing of their expertise concerning mental illness [10, 11].

In terms of health research, it has been long recognised that evidence is needed to support the use of methodological processes with priority setting, as well as the procedures involved to identify the studies [12]. Yet understanding their use with mental health research remains underexplored [13]. Potential reasons for this are a propensity for priority setting to generate and report on priorities rather than the methods to obtain the results, and lack of funding compared to other areas of healthcare suggesting this too impacts on what priorities are decided [14, 15]. In a study of European countries, the share of funded health research dedicated to mental health ranged from 4·0% in the United Kingdom (UK) to 9·7% in Finland [16], while Woelbert et al. [17] noted a flat and stable trend in funding over the years 2015–19 and unequal geographical distribution. Even with underfunding, the obligation to prioritise mental health research cannot be overstated. Over 1 billion people are affected by mental disorders globally, bringing about 7% of all global burden of disease with 19% of all years lived with some incapacity owing to mental illness [18].

No consensus on the optimum model for best practice appears to exist, or what constitutes high quality in developing priorities for mental health research despite growing mental health problems [14]. This is a knowledge-gap that requires attention given the efficacy of priority setting is “determined by the use of systematic, explicit and transparent processes to increase research funding” ([8], p.2), while funding for mental health research is disproportionate to other health topics. Methodological procedures are preferably evidence-based to be a vehicle to generate robust results, since mental health research requires to have the greatest potential public health benefit while proficient and fair with use of constrained resources [8, 12, 13]. Explicit procedures may also contribute to an inclusiveness of different voices within projects, rather than the tradition of only academics deliberating what research is prioritised. Namely, patients and their significant others who are ultimately impacted by the changes from research, while procedural transparency can help these groups to assess the rigour in how research was prioritised [10]. To that end, procedural knowledge that contributes to effective priority setting is essential, and to date, there appears no comprehensive review in what approaches can be adopted and why, with prioritising mental health research [13].

## Rationale for review

Given the factors involving underfunding and burden of mental illness, it is important that priority setting adopts evidence based approaches to identify research with value and impact. In keeping with such conscientiousness, the review aim was to summarise methodological procedures located within current and relevant literature identifying mental health research. Hence, provide a flexibility and critical guide of methodological procedures available for mental health stakeholders who wish to undertake a prioritisation project. The review was supported by a preliminary search of databases such as the Cochrane library^1^ to ensure that a litrature review covering the same topic was not published in some form. Adopting the definition of priority setting as the targeting of research with potential public benefit [19]; the central question and sub question of the review were as follows:

1. What methods, designs and frameworks are implemented with priority setting mental health research?
2. What are the characteristics and purposes of these methodological procedures?

Since the field appeared underexplored, the objective was also to locate and critically evaluate the methodological procedures employed with prioritising mental health research, to inform the discussion about the considerations for future projects later in the paper.

## Methods

A systematic review of published literature was selected as the best method to address the review questions, in terms of providing a structured process that limits selection bias and generates reliable results [20]. The latest PRISMA guidance was followed to ensure accurate reporting and rigour in the process of identifying and analysing literature [21, 22]. A review protocol was not published on Prospero^2^ as standard practice is not to publish a protocol without patient outcomes; however, the originality of the review was supported by the aforementioned preliminary search.

### Search strategy

Frameworks and designs were defined as pre-existing guidance or a methodological approach informing the overall priority setting process, while methods were steps to achieve pertinent stages of prioritisation, such as ranking of priorities [12]. Mental health was defined in terms of psychological and emotional wellbeing or degree of lacking these when involving illness [23].

An initial search between 1st July 2020 to 1st November 2020 identified papers limited to scholarly and peer reviewed journal articles for the time period of 1st January 2012 to 1st July 2020. A subsequent search in January 2022 updated the results of papers published between 1st July 2020 to December 31st, 2021, to ensure contemporary findings and the reviewed literature was from the last 10 years (2012–2022). A senior university librarian provided guidance to develop the accuracy of searches, while the following health and social care databases were searched as these potentially hold relevant papers: The Allied and Complementary Medicine Database; CINAHL Plus; MEDLINE; APA PsycArticles; Applied Social Sciences Index and Abstracts; International Bibliography of the Social Sciences; PTSDpubs; Scopus and Social Policy and Practice.

The full text of papers within databases were scanned in case that the abstract or title did not contain the key search terms [24], while Boolean Operators (AND/OR) were employed to generate search term combinations and Truncations [*] to find variations of the root of a word to expand the search. The following keyword combinations were searched: [“mental health” OR “psychiatry” AND “research priority setting”], [“mental healt*” OR psychiatr* AND “resear* priorit* Sett*”] and [“mental health” AND “decid* sett* AND “resear*”].

### Inclusion/exclusion criteria

All retrieved papers were screened for eligibility against the inclusion and exclusion criteria in Table 1. To not limit findings, there was no exclusion of papers based on priority setting participants or priority setting topic if following the aforementioned definition of mental health. International papers were also accepted in view these may expand the identification and knowledge of methodological procedures adopted with priority setting, though the papers required to be written in English to ensure the literature could be understood.

**Table 1:** Inclusion and exclusion criteria

Inclusion	Exclusion
Last 10 years	Theoretical discussions or opinion papers
In English	Letters
International literature	Protocols
Peer reviewed	Research not involving mental health
Clearly sets out methodological procedures assessed against REPRISE	Developmental disorders

### Data extraction

**Fig. 1:**
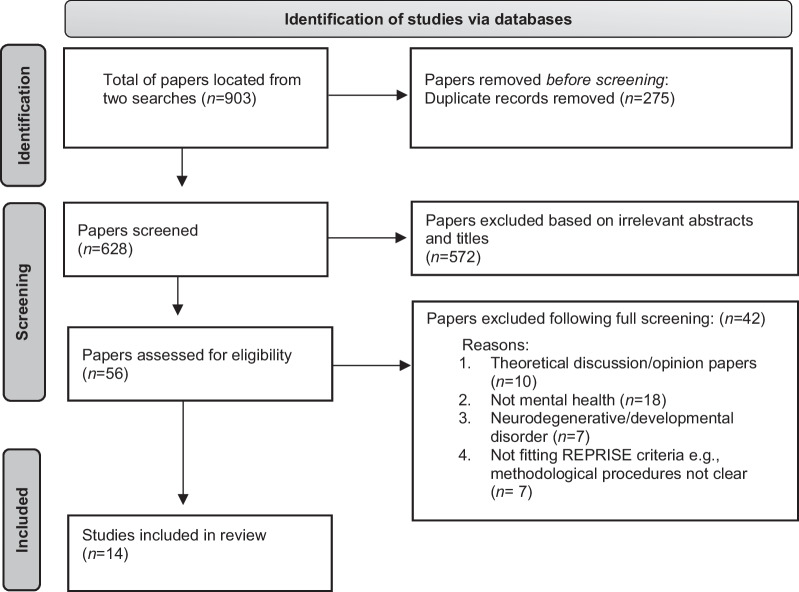
PRISMA flow diagram of the article search process

For both searches, two researchers (K.D. and J.C.M) separately considered all papers for inclusion, discussing any discrepant views together with a third researcher (N.B) to reach a consensus. Identifying papers involved removing duplications through an automated process, then the two researchers (K.D and J.C.M) excluding irrelevant titles and abstracts. The full screening of the remaining papers included checking independently that methodological procedures were clearly explained and present in the articles (K.D, L.R, J.C.M and D. A.C.). To aid this process, recommendations by Tong et al. [25] The REporting guideline for PRIority SEtting of health research (REPRISE) were followed, and this involved checking if the papers (1) demonstrated the aim of priority setting; (2) highlighted the recruitment strategy; (3) illustrated the participants and (4) presented descriptors of methods. See Fig. 1 for a PRISMA summary of the filtering process.

### Quality appraisal

Despite the apparent paucity of frameworks specifically designed to evaluate the quality of priority setting procedures, an assessment was undertaken to inform the considerations for priority setting section later in the paper, while such appraisal is an expected component of PRISMA guidelines [21, 22]. Priority setting procedures may vary greatly from research methodologies and methods [26]. This can diminish the accuracy of the appraisal using tools to evaluate research, for example the Critical Appraisal Skills Programme (CASP) [27]. However, a critical interpretive synthesis informed the analysis whereby the appraisal of methodological procedures integrated into the synthesis of the findings [28]. To promote objectivity, the critical synthesis also adopted the categories from the good practice checklist by Viergever et al. [12] as recommended by Mador et al. [26], explained in further detail below.

### Data-synthesis

A convergent qualitative design was employed to transform results into a qualitative format, with the method reporting statistics using words rather than figures. This allowed for heterogeneous results to be synthesised into the same review [29]. The synthesis was informed by abduction, involving the interplay of deduction and induction. Inductively, the checklist by Viergever et al. [12] guided what constituted methods, designs, and frameworks to find, while induction involved locating these within the priority setting literature selected for the review. The last step was categorising the methodological procedures located using a spreadsheet with columns advised by the checklist, adding rigour to the synthesis by applying a reliable approach to shape the critical outline of findings.

Not all nine categories were utilised from the checklist by Viergever et al. [12], notably actions following priority setting were omitted as not seen relevant to the review. In addition, the research team discerned that several categories from the checklist could be amalgamated for the purpose of the critical synthesis, involving: (1) *Comprehensive Approach*—frameworks/designs guiding the entire priority setting, including preparatory work, and reasons for the project; (2) *Inclusiveness*—participation methods; (3) *Information Gathering*—data collecting methods to identify research gaps, and; (4) *Deciding Priorities*—methods involved with finalising priorities [12].

## Results

The findings section outlines the key review results. The characteristics of the priority setting are provided before presenting the main findings synthesised through the good practice checklist. Table 2 presents a summary of the forthcoming synthesis highlighting the typical methodological procedures found tabulated through the four checklist categories.

**Table 2:** Typical methods, designs and frameworks identified within prioritisation projects

1. Comprehensive approach	2. Inclusiveness	3. Information gathering	4. Deciding priorities
Focus on a participatory approach	Use of stakeholder steering groups involving some patient representatives	Review of the literature (research, policies, and guidelines)	Tended to identify research themes and questions
Stakeholder steering group set up to inform prioritisation agenda	Stakeholders appeared recruited contingent on how their expertise aligned to the aims of the priority setting	Online surveys	Nominal group technique to finalise priorities
Modified James Lind Alliance framework	Attempts made to have some patient and caregiver participation with identifying priorities	Focus groups	Ranking (including metric-based) of priorities
Modified Child Health and Nutrition Research Initiative framework	Recruitment involved advertising via social media, gaining contact using databases of relevant stakeholders, and with assistance of advocacy groups	Modified Delphi exercises	Collaborative workshops
Systematic mapping review to inform prioritisation agenda Literature review to inform prioritisation agenda	Mostly researcher led with designing processes and overseeing key phrases	Nominal group technique	Combining consensus building in group work e.g., use of nominal groups
Consensus building using the Delphi method		Online consultations	Consideration given to consensus building with finalisation of priorities
Stakeholder surveys to inform prioritisation agenda		Consultation with advocacy/patient groups	Interactive voting techniques such as “dot-mocracy” [36:2]
Discursive group meetings to inform prioritisation agenda		Workshops/discursive meetings	Combining ranking and discussion within group work
Consultation with advocacy/patient groups to inform prioritisation agenda			

### Priority setting characteristics

Thirteen priority setting projects were described in fourteen separate papers (two of the fourteen described the same project and therefore used the same project) [30, 31]. Priorty setting projects were conducted in the United Kingdom (*n* = 3) [32–34], Australia (*n* = 3) [35–37], Canada (*n* = 2) [30, 31], Canada, Sweden, United Kingdom, and the United States (*n* = 1) [38], Brazil (*n* = 1) [39], Chile (*n* = 1) [40] and Germany (*n* = 1) [41]. The remaining two papers, one described prioritisation to develop a Roadmap for Mental Health Research in Europe (ROAMER project) [42] and another developed priority areas across humanitarian settings in low and middle-income countries [43]. Mental health disorder-specific priorities were identified for depression (*n* = 1) [30, 31], depression and bipolar disorder (*n* = 1) [35], eating disorders (*n* = 1) [37], obsessive–compulsive disorder (*n* = 1) [41] or broadly for long-term conditions for older people (*n* = 1) [38] and mental health in terms of dementia [33], while research was prioritised for psychosocial interventions in areas of humanitarian need (*n* = 1) [43].

### Critical synthesis

The following is the synthesis of findings informed by the checklist categories. Focus is on the variable ways methodological procedures were employed to guide priority setting projects, while a more detailed account of methods, design and frameworks is provided in Table 3.

**Table 3:** Priority setting characteristics

Paper	Location	Participants	Aim and purpose	Guiding framework/design	Methods	Outcome
Aboaja et al. [32]	United Kingdom	Patients from both prison and forensic hospital settings (no numbers available)	Involve patients with research priorities in forensic services	Consensus methodology using the Delphi method	• Information gathering ascertaining patient priorities in phase 1, ranking these in order of importance in Phase 2: • Community meetings chaired by a senior nurse (who collated data) guided discussions around three questions: 1. What questions should researchers try to answer? 2. What discovery would you most like researchers to make? 3. What do we need to know more about in forensic services? • Analysis conducted overall by two consultant psychiatrists, in-patient nurse, ward-based staff nurse and a senior prison nurse	Top 3 research gap themes
Banfield et al. [35]	Australia	Self-identified mental health ‘consumers’ (*n* = 50)	Explore patient priorities for depression and bipolar disorder research	None identified	• Focus groups with patients and telephone interviews with consumer advocates • Participants discussed topics believed were priorities • Priorities of top 3 ideas ranked • Transcripts were thematically analysed using NVivo 7	16 themes of research gaps without apparent consensus on top 3 priorities
Banfield et al. [36]	Australia	Forum: Patients (termed consumers) (*n* = 14), carers (*n* = 5), carers and patients (*n* = 5) Online survey: Consumers and/or carers (*n* = 70)	Determine priorities for research driven by the views of consumers and carers	None identified	• Face-to-face forum, then national online survey • Forum participants developed topics for research in small group discussions and then voted on priority using a “dot-mocracy” approach—placing adhesive dots to vote for topics seen important for research • Online survey developed from the voting. Participants were asked to rate these topics on a 5-point priority scale and rank the importance of the highest-rated topics	87 research themes identified without apparent consensus on top 3 priorities
Breault et al. [30, 31]	Canada	Steering group: people with depression (*n* = 6), caregiver (*n* = 1), clinicians (*n* = 4), researchers (*n* = 5), and members of the Alberta Depression Research Priority Setting Project planning committee (organisation leading the priority setting) (*n* = 2) Online and paper survey: respondents with depression (*n* = 445) Online rating: respondents (unclear if these had depression) (*n* = 49) Workshop: Steering group members (*n* = 11), clinicians, health care professionals and people with depression (*n* = 9)	Identify patient research priorities involving most unanswered questions about depression	Informed by the James Lind Alliance (JLA) approach and “funnel approach” [31:E399]	• 6-step funnel process to gather and prioritise questions facilitated by a steering committee: - 1. Online and paper survey 2. Open-ended questions reviewed by steering group 3. Online rating survey using a 5-point Likert scale 4. Workshop to identify the top 10 questions 5. Literature review of top 11 questions regarding originality 6. The final report was shared through the media	11 research questions, unclear if ordered in priority but are listed 1–11
Chamberlain et al. [38]	Canada/Sweden/United Kingdom/ United states	Online survey: distributed to members of Voices Of Individuals, family and friend Caregivers Educating uS (VOICES) involving Researchers (*n* = 40), health stakeholders (*n* = 20), and citizen advisory committee involving those living in residential care, living with dementia, alongside family, friends and caregivers (no numbers) Workshops: non-researchers (no further data available)	Identify research questions within existing Translating Research in Elder Care Program data	Informed by JLA approach	• Online survey analysed by researchers • Survey data examined in workshops using Nominal Group Technique (NGT) and sequential ranking to home in priorities	10 research questions unclear if ordered in priority but are listed 1–10
Emrich-Mills et al. [33]	United Kingdom	Patients aligned to the healthcare organisation leading the priority setting, including family, friends or other caregivers, and clinical staff Anonymous online survey: respondents (n = 126) Second survey: respondents: (*n* = 58) Workshop: participants: (*n* = 26)	Identify patient and staff research priorities to inform funding applications to address local needs	Informed by JLA approach	• Anonymous online survey to collect research interests from stakeholders then categorised electronically • Systematic search of the literature was conducted to assess the originality of questions, following PRISMA guidelines • Second survey ranking top ten using 6 different versions with different ordering of questions, grouped relevant to the organisation • Questions were reviewed in the workshop using NGT and sequential ranking to home in priorities	10 research questions ranked in order of priority
Forsman et al. [42] [addendum paper by Fiorillo et al. [48] highlighted the participants involved]	Europe	Mapping of research, workshops, and emails: Published scientific experts (*n* = 29) Surveys and workshops: renowned researchers (*n* = 59) and other experts (*n* = 44). The project also involved psychiatrists (*n* = 31), other mental health professionals (n = 30), patients/carers (*n* = 23) and trainees *(n* = 20) [48], though unclear if this sample is the same as above	Develop standardised measures to investigate ranked determinates of mental health	Roadmap for mental health research in Europe project method (ROAMER)	• Mapping of published research advances, to identify developments and gaps over the last 10 years • Workshops and emails from participants identified questions These were grouped in terms of effectiveness, deliverability, and feasibility • 3 × modified Delphi web-based surveys (DWBS) were conducted sequentially, to gain consensus on research priorities DWBS 1—Assessed relevancy of questions DWBS 2—Ranking based on general, methodological and research topic priorities • Second workshop to home in priorities. based on the criteria of the second Delphia survey DWBS 3—Final survey ranked themes	• Summary principles generated in terms of informing mental health research • Ranking of priority themes • Themes categorised to specific goals to aid societal mental health
Ghisoni et al. [34]	United Kingdom	Workshop: mental health professionals (*n* = 5) and people with mental ill-health (*n* = 20), some also carers (*n* = 3)	To explore research priorities from the perspectives of people with lived experience of mental illness, their carers, and clinicians	None identified	• A qualitative NGT employed • Workshop to introduce exercise • ‘Brainstorming’ at 6 Workstations exploring pre-existing themes • Ideas condensed on a flip chart • 3 rounds of voting using workstation themes, preferred research according to patients and carers, feasibility voting of research that could occur now and be funded	One research question received most votes while another question was noted as most discussed
Gregório et al. [39]	Brazil	Researchers (*n* = 22) and policymakers (*n* = 5)	Review the agenda for research priorities	Informed by Child and Nutrition Research Initiative (CHNRI) approach	• Participants list on their own the most relevant research questions for the next 10 years. Responses were distributed following predefined research areas informed by policies and a literature review (not conducted by the priority setting group): 1. Improve in place health systems 2. Improve the efficiency of health systems in place focusing on health policy and systems 3. Improve affordability and deliverability of existing interventions 4. Develop new mental health interventions • The most significant questions were ranked • Questions scored addressed Answerable, Effectiveness, Deliverability, Equitable, and Reducing mental illness • Ten highest ranked questions were selected	10 research topics ranked in order of priority
Hart and Wade [37]	Australia	Eating disorder specialist (n = 103), patients/carers (*n* = 109) and affiliates (*n* = 53)	Consensus building among clinicians, researchers, carers, consumers, and interested members on the priorities for eating disorders research	Referred to as a Delphi study	• Employed Delphi expert consensus • Online survey—Qualtrics—rating research areas, and research domains in order of priority. National Collaboration for Eating Disorders (NEDC) provided areas for priorities • Leading scientist in the field met to review for research areas that could be priorities, these were then added • Research areas are grouped into domains according to thematic analysis • The first-round survey involved ranking seven broad research domains in order of priority for consensus on the top funding priorities	22 ‘essential’ themes ranked in order of priority
Kühne et al. [41]	Germany	Email survey: Clinicians (*n* = 8) Internet-based survey: patients (*n* = 63)	Meta-review and survey of professionals and patients concerning psychotherapy and obsessive–compulsive disorder (OCD) research priorities	Informed by JLA approach	• Meta-review of databases re-aim • German S3-Guideline references on OCD searched with 19 reviews identified • 10 priorities were generated from the discussion of reviews and then phrased as a question for subsequent surveys • Email survey. 5 most important priorities identified following open-ended question to identifying priorities, ranking each (clinicians) • Internet-based survey. Open question regarding 5 top priorities and then ranked the 10 identified previously (patients) • Non-parametric tests to compare agreement	Top 5 Patients and clinicians views ranked owing to similarities only one top 5 list produced
Lee et al. [43]	Global settings	Generating terms of reference: Psychosocial support (PS) in humanitarian settings stakeholders (*n* = *109*) Online survey: Same stakeholders (*n* = 87)	Consensus building on psychosocial support (PSS) interventions using evidence and stakeholder opinions	None identified	• Mixed methods with recording discussion, voting, and statistical ranking • Phases involved individual consultation • Expertise consisted of; 2 regional meetings, 4 webinars, and a series of priority setting exercises Process: Briefing on project focus—no specific operational definition for interventions Stakeholder engagement to generate terms of reference for priority setting A systematic review to identify the research area NGT to develop a priority PSS program list Online survey to rank priorities NGT to further priorities outcomes alongside ranking Webinars / Series of priority setting exercises and Consultation and agreement across stakeholders to agree on the priorities Individual consultation involving steering group to gather recommendations for research topics Concluding online survey to vote on priorities	16 programs/areas relevant to PSS through voting frequency counts without apparent consensus on top 3 priorities
Zitko et al. [40]	Chile	Initial interviews: Members of the Ministry of Health (*n* = 6) Focus groups: Three in total, one with representatives of patient groups (*n* = 6), second members of the National Commission for the Protection of Persons with Mental Disorders (*n* = 5), and the third clinicians (*n* = 7) Online consultations: academics *(n* = 63) Final prioritisation: consultants (*n* = 2), epidemiologist (*n* = 1) and psychiatrist with public policy training (*n* = 1)	Identify and prioritise national research gaps	Informed by CHNRI approach	• Stage 1: 4 × strategies to identify knowledge gaps document analysis, interviews and focus groups, alongside online consultation of the scientific community • Stage 2: Elaboration and application of prioritisation criteria for ranking each research question in terms of the knowledge gap, breadth of the relevant population, their vulnerability, potential risks and benefits, information urgency and application	10 questions relevant to government policy ranked in order of priority

#### Comprehensive approach

The first category explores frameworks/designs guiding the priority setting including preparatory work, and underpinning reasons for the project. Raising the profile of mental health research (e.g., Aboaja et al. [32]) and exploring the use of finite resources for service provision (e.g., Zitko et al. [40]) were common motives to conduct priority setting. However, while limited resources for mental health research, and generating research suitable for funding appeared to be reasons for the projects, no project limited their final priorities based on the rationing of research costs. Alternatively, the majority aimed to document patient and healthcare professional views to inform future research agendas, while two individual projects confined their evaluation to eliciting patient views alone [32, 35].

The use of frameworks and designs to guide priority setting was limited, though demarcation existed between aiming to promote public involvement, such as identifying patient and caregiver informed research, and health policy approaches to deciding investment priorities. The latter focused specifically on reducing disease burden and inequity [35, 39, 40, 42]. Aboaja et al. [32] and Hart and Wade [37] employed a modified Delphi approach for their priority setting design involving rounds of questions discussed in groups, then aggregated to reach consensus [44]. Well-known frameworks for priority setting were identified, notably the Child Health and Nutrition Research Initiative (CHNRI) and the James Lind Alliance (JLA). Defined as an interpersonal framework to build consensus, the JLA aims to generate a top 10-priority list [45] and four projects used the JLA approach [30, 31, 38, 41].

The CHNRI employed by Gregório et al. [39] and Zitko et al. [40] was based on determining five components: population, disease burden, geographic limits, timescale, and investment [46]. To fulfil this brief, projects using the CHNRI recruited subject and scientific experts alongside advocates, mid-level implementers and key, strategic, decision-makers at policy level to inform national priority-based resource allocation agendas [39, 40]. When the JLA and CHNRI were applied, modifications were made to both frameworks. Attempts were made to improve quality and suitably accommodate the parameters of specific projects, by augmenting structured stages with additional processes and tasks. For example, Breault et al. [30,31:E399] added two additional stages to the JLA partnership model referred to a “funnel approach” to channel patient participation and home in on the generating questions. Conversely, other projects were inspired by the frameworks but omitted key phases of best practice due to what appeared to be a limitation with resourcing [33, 41], or making use of existing data [38].

The JLA [47] suggests that final priority lists have an existing, adequate evidence base to support adoption and implementation, and comprises of the extensive reviewing of the literature alongside expert checking. This phase appeared omitted by some of the selected projects in the review [34–38, 42], and may reflect a process issue whereby the finalised priorities are not sufficiently supported by the evidence base [12]. Two papers suggested that using experts as participants justified not checking whether research existed to answer identified questions [39, 40]. However, the researchers focused on ensuring contextual relevance of the final list of priorities by utilising existing policy documents to shape key informant’s discussions in the initial information gathering stages. For example, Zitko et al. [40] analysed clinical guidelines and national health strategies to identify specific research questions for prioritisation, while Gregório et al. [39]. directed key informants to guide their deliberations using a national clinical strategy.

International priority-setting projects performed more robust, systematic mapping and syntheses of existing evidence for prioritisation. It is unclear whether systematic mapping influenced the development of priorities in the ROAMER project [42] though reference to other work packages to document the perspectives of patients, carers, clinicians, and policymakers suggests the researchers aimed to develop a harmonised research priority agenda [48]. Similarly, in setting global priorities for humanitarian interventions, Lee et al. [43] considered these complementary processes, inviting 160 key (*n* = 109 accepted) informants for individual consultations to ensure that the information gathered represented international perspectives on important research areas.

#### Inclusiveness

Inclusiveness identifies participatory methods to aid joint decision-making, and whilst few papers reported operationalised objectives underpinning the methods selected; the majority adopted participatory methods of some form stressing the importance of stakeholder involvement in determining priorities. However, it was not clear how all participants were recruited in some projects [30, 35, 39, 42, 43, 48], although in other projects stakeholders were contacted using databases or patient data held by the lead organisation [32–34, 38, 41] or relevant advocacy groups [36–38], and social media advertising [30, 31].

The aim of the priority setting appeared to impact on participant selection, notably to promote patient involvement and identify their views about beneficial research [30–38, 41, 42], alongside draw on more traditional expertise involving researchers and clinicians [33, 37, 39–42]. In other projects, ‘users’ were considered as a range of stakeholders of healthcare research and in some included patients, caregivers, and healthcare professionals [30, 31, 34, 35, 41] and in others, wider groups included advocates, managers, and administrators [35–38]. The extent these priority setting projects enlisted stakeholders to define the parameters of the exercise and mobilise their own communities to produce priorities varied substantially. All except two exercises [35, 36], were initiated and led by researchers. Some engaged patients to comment on processes [33, 36], appointed steering groups comprising of patients, advocates, professionals, and academics [34, 37], or developed partnerships who assumed responsibility for key decisions such as deciding on the scope and overseeing the conduct of successive phases of the projects [30, 31].

#### Information gathering

The following examines the methods to collect relevant data such as research gaps to determine priorities. A mixture of online surveys [30, 31, 33, 37, 38, 41, 42], structured group discussions [32, 34–36, 40, 42], stakeholder engagement and systematic review [43] alongside individual participants listing research gaps [39] were used to develop initial key questions/topics that needed to be addressed by research. These were prefaced with evidence-based knowledge of emerging research areas, meta-reviews, or existing available databases in some projects [37, 38, 41] to inform the development of surveys.

Information-gathering methods within priority setting included qualitative focus groups—assembling participants to discuss priorities [35, 36], nominal group technique (NGT)—structured small-group discussions involving deliberating and voting [34, 43, 44] and modified Delphi exercises [32, 37, 42]. Group discussions were used to bring stakeholders together to identify priorities in some approaches [32, 34–36] two of which generated and ranked priorities at the same meeting [35, 36] and one used existing patient community meetings within hospitals [32].

In addition to the three consensus building methods described, surveys and online consultations were also used. One project engaged members of a steering group to codesign questionnaires [31], one engaged researchers and patients [33] and in another, researchers worked with wider advocacy or patient groups [37]. However, one project designed the survey without stakeholder participation though it was evidence-informed in which priorities were cross-referenced with the literature [41]. Measures were taken to enhance the relevance of survey questions to potential participants including providing examples and definitions of research [30, 31, 36], categorising research areas in advance of the survey [33, 37, 38], and utilising evidence and policy to inform the design [37, 39, 41]. However, no projects reported piloting or refining the questionnaire before commencing the survey.

#### Deciding priorities

The last section considers the methods to finalise priorities presented in two-parts; refinement/ranking and finalisation of priorities.

##### Refining and ranking generated priorities

The key task of refining stakeholder-generated priorities is formulating questions that conform to searchable frameworks while retaining the intended meaning of the respondent. In some instances, projects sought to identify thematic areas of topics that circumvented the need to identify specific questions [32, 35, 37] and were derived through qualitative analysis of responses, such as workshops [30, 31], including “dot-mocracy”, using adhesive dots on a flipchart to vote for research topics ([36], p. 2). Other refining methods involved online surveys [33], ranking [39, 41], and expert analysis without patients [37, 40]. Metric based ranking and obtaining final priority lists were also merged into one exercise in some projects such that ranking and gaining consensus was merged into one activity, e.g., Forsman et al. [42].

##### Finalising priorities

Group consensus approaches were used in several projects, although as highlighted priority metric-based ranking was also employed which resulted in final priority lists [32, 36, 42]. The outcome for priority setting included valuable lists of research gaps without necessarily agreement on which should be prioritised [35]. In other projects, respondents identified their top three priorities and frequency counts were obtained, without always explaining whether these responses were weighted. Aboaja et al. [32] identified weighting of 10.7% with patient responses, whereas Breault et al., [30, 31] provided little detail in terms of responses though presented the demographics of the participants who responded. Ranking of priorities also varied, with several projects distributing successive phases of ranked data for further refinement based on sophisticated criteria [39, 42, 43], while one project took a percentage of endorsements of broad research priorities [37]. Collaborative workshops based on consensus-methods were also utilised, employing NGTs or adapted versions of these [30, 31, 33, 38] which are strengthened by the iterative nature of gaining consensus on priorities through active discussion and participation. However, only one project selected a top research priority using participant voting in workshops [34].

## Discussion

Priority setting frameworks predominately employed within the sample of fourteen papers were the JLA and CHNRI. The JLA was the most used although often in modified form, and whilst not always clear as to why these adaptions occurred, Kühne et al. [41] reported this was owing somewhat to financial constraints. Not only can cost potentially impact on the way frameworks are adopted but also patient involvement, notably Boivin et al. [49] identified a 17% increased cost for involving patients, suggesting such stakeholders can be priced out of participation. The other notable framework identified was the CHNRI, also modified, with apparent focus on some of its categories to collate research topics involving symptomology, illness burden, equality, and budgetary impact [39, 40]. Some papers did attempt to explain adaptations made to frameworks by signposting to other articles, although not necessarily fully clarifying the reasons for changes. Amongst motives for such signposting, may involve ‘Salami Slicing’ whereby the project is published over several articles to increase citations, lessening understanding of methodological procedures, as not presented as a cohesive whole in one article [50].

In addition to the JLA and CHNRI frameworks, it was also found that the papers used two objectives to inform the priority setting projects:

A. Generate research topics in terms of available or limited resources, for example the affordability of research [39], efficient use of limited research funding [37], the cost effectiveness of research [40] and/or
B. Capture the voices of living experiences, for example, from patients and caregivers to inform care [36].

Barra et al. [51] characterises these two points as a likely politicising amongst stakeholder views between generating meaningful research and research rationing, given finite resources. Rationing, in terms of identifying research based on cost effectiveness alone was not overly apparent, though as point A. highlights, rationing of mental health research in some way was reason to why some projects occurred. Hence, when influenced by what can be realistically funded, a politically charged terrain does seem inescapable, especially as such restrictions may potentially shape priorities not necessarily addressing patient concerns, or insufficiently substantial to initiate policy changes that improve mental health [14].

Methodological procedures in the papers were also found to be somewhat directed by the priority setting aim. Preferences included consensus building, particularly when the aim was to enrich the patient voice, symbolic of going to the heart of mental health care involving coproducing knowledge through some interpersonal connection [52]. These resonated with democratic group methods such as the NGT to ensure all voices were heard, but also, not necessarily concurrently, discursive methods like qualitative focus groups, at times involving policymakers and budget holders when seemingly tied to seeking value for money to inform national policy [40]. Some projects ranked and engaged in discursive exercises to gather uncertainties simultaneously, e.g., Forsman et al. [42]. Whilst the approach may lessen the dominance of individuals and reduce cost, it could result in a common representation of research priorities to ensure participants make an agreement. This could impact on the originality of the priorities, and without necessarily addressing a knowledge gap, may limit the implementation as a research project [53].

Several groups recruited for the priority setting projects appeared to represent the target stakeholder population, whilst the recruitment process of other projects lacked clarity. For example, priority setting considered the mental health of young people [34, 42, 43], though the reporting of involving young people as participants was not clear, and if not involved, suggests a possible disparity with prioritising research enlightened by the views of children and adolescents. In general, greater opportunities for participation existed for those from professional backgrounds, raising philosophical questions in what constitutes expert knowledge with some priority setting projects [54]. Professionals such as policymakers and scientists may have better vantage points given their expertise and experience about the feasibility of priority setting and ways to reach the endpoint of funded research [55]. Cost of research training might also have implications about who can participate [49]. However, living experiences of care are attributes to identify meaningful research topics, signifying the importance of patient and caregiver views, and whilst training cost is an issue, it may simply involve raising awareness about the parameters of prioritising research to ensure its success [56, 57].

Not all projects started with a clear scope or terms of reference. Whereas some commenced with literature reviews, systematic mapping reviews were an alternative. Although use of review mapping in one project was unclear in how it impacted on the priority setting process [42], the method can aid prioritising by mapping research gaps within a given research field, providing further evidence to implement the identified priorities as research projects [58]. While bringing about an evidence-informed approach, identifying priorities from available databases or research may narrow patient choices. Final priority lists could potentially omit research areas that are both important to patients and neglected by research reducing the potential impact of priority-setting project to address gaps in the evidence base. Alternatively, different forms of surveys were adopted to commence priority setting, drawing on wider and on occasion more ambiguous research terrain.

Overlooking the gaps and needs of the research field, makes priority setting difficult to achieve [25]. Reviewing the literature suggests that a pragmatic approach is needed in preparation for a prioritisation project, to improve its focus with mapping out research gaps, but combined with gathering diverse expertise, such as from patients when concerning care, to improve the understanding of research needs [47]. This appeared within a contextual focus concerning particular mental health conditions or other relevant care factors aligning to the participant expertise. For example, when seeking to make use of resources in some way, budget holders appeared more recruited for priority setting projects [40].

Having a clearly defined aim is likely to help inform the methodological procedures to be taken in a prioritisation process. The aim should take account of the complex context, including funding, resources, and feasibility and other factors influencing mental health research [3, 47]. Clear and precise project aims may be less likely to produce broad themes that appear too ambiguous to be financed [26]. Given the limited research funding available, methodological procedures must be such that the endpoint of priority setting are research topics that easily translate into actual investigations.

Although themes might not always convert well into specific research projects, limitations with funding also play a role in skewing research priorities towards those involving hypothesis testing. This may not always correspond with what patients find useful, for example, understanding experiences of care to develop practice [59]. Despite the aforementioned risk of politicising, without taking funding into consideration, priority setting might give the impression of appearing superfluous if not leading to substantial investigations. When involving patients, priority setting in such circumstances could appear tokenistic, and reaffirm a sense of underrepresentation, by patient views not transforming into actual research projects [56]. The same could be proposed with lists without obvious ranking, suggesting a further step is required to home in on a specific priority, in consideration of the competitiveness, and limited funding available for mental health research. The JLA [47] somewhat echoes this view, in which priority setting results in the top 10 priorities in order of importance.

### Considerations for priority setting

The critical analysis of priority setting procedures seems a fledgling field. However, the checklist by Viergever et al. [12] not only supported the synthesis of findings, but alongside the discussion of the paper, helped to develop the following considerations to inform future priority setting projects specific to mental health research.

A. Priority setting appeared beneficial when involving a range of expertise, as highlighted by Foresman et al. [42], aligning patients, scientists, and policymakers to subgroups in which they may have greater knowledge, while subgroup views were reviewed by other participants [42]. Given priority setting may examine mental health concepts that are broad in nature, the above approach might be considered for it allows a deep dive into specific parts that make up the vast mental health field under exploration [60].
B. Despite the review highlighting inclusivity of patients and caregiver views, there was little evidence of co-producing the priority setting project with these participants. Hence suggested is that such involvement improves to enhance the identifying of research relevant to those in receipt of care and their significant others.
C. The papers reviewed invariably reported the adoption of recommendations or good practice guidance such as Viergever et al. [12], and given the importance of rigour with identifying priorities, such guidance is ideally utilised to shape the priority setting project.
D. When adapting frameworks for example as provided by the JLA, consideration is given to these adaptions as part of writing up, alongside stating why these adaptions were made. This can help to understand methodological congruence, and although predominately applied to research, the WHO [7] alludes to the approach when planning the coherence of projects, so that the priority setting aim(s) aligns to the purposes amongst its methodological parts. Thus, provide the rationale for adaptions and why methods were employed, also acknowledging the shaping of methodological procedures via limitations such as funding and feasibility [25].
E. The aim(s) and approach of the final research priorities needs to be explained to aid their funding. Priorities otherwise may not develop into research projects and may reaffirm that some participants are less likely to have their voices heard, notable with patients [61].
F. Given the diversity of mental health research, the final consideration is for priority setting to go beyond only illness. Problematising mental health appeared evident with the literature, loosely tied to mental illness and mental health problems. Research about mitigating illness may receive more funding over maintaining and promoting mental health [10]. However, consideration should also be given in how research can enrich the lives of people, so they may thrive and thereby lessen the prevalence of mental health difficulties [62, 63].

### Review limitations

The review was limited by challenges with identifying search terms for prioritisation, which potentially may have excluded papers otherwise meeting the inclusion criteria. The lack of a standardised approach to the critical appraisal was also a limitation, for such appraisal is the cornerstone of systematic reviews to assess the quality of investigative methods and inform the direction of future research [20]. However, to apply a critical approach, the review drew on the seminal work of Viergever et al. [12] to guide the synthesis and inform the above considerations. Whilst perhaps not providing the depth of critique such as employing the CASP [27] with reviewing research, a recognised approach was nevertheless utilised to identify and review the methodological procedures located within priority-setting projects.

## Conclusion

This systematic review summarised frameworks, designs and methods adopted with priority setting for mental health research, to inform stakeholders in mental health about the methodological procedures to conduct priority setting, be it from grassroot levels to more national approaches. The findings highlighted that while a growing trend with involving participation from experts by experience such as patients, there is room to improve their leadership roles where feasible. Prioritisation frameworks, notably the JLA and the CHNRI were utilised but were adapted in practice, potentially impacting on methodological quality. Generally, greater clarity in defining the aims of priority setting would support the appropriate selection of methodological procedures that may lead to the creation of actual research projects.

## Data Availability

The datasets used and/or analysed during the current study are available from the corresponding author on reasonable request.

## Footnotes

1 The Cochrane Library is a database containing high-quality health related systematic reviews https://www.cochranelibrary.com/.

2 The International Prospective Register of Systematic Reviews (PROSPERO) is database to register a systematic review protocol to raise awareness of the work and limit duplications https://www.crd.york.ac.uk/prospero/.
